# Efficient Extraction of Deep Image Features Using a Convolutional Neural Network (CNN) for Detecting Ventricular Fibrillation and Tachycardia

**DOI:** 10.3390/jimaging9090190

**Published:** 2023-09-18

**Authors:** Azeddine Mjahad, Mohamed Saban, Hossein Azarmdel, Alfredo Rosado-Muñoz

**Affiliations:** GDDP, Department Electronic Engineering, School of Engineering, University of Valencia, 46100 Burjassot, Valencia, Spain; mjahad.azeddine@uv.es (A.M.); mohamed.saban@uv.es (M.S.); azarmdel@alumni.uv.es (H.A.)

**Keywords:** Biomedical Systems, Electrocardiographic Signals, ventricular fibrillation, ventricular tachycardia, time–frequency representation, non-stationary signals, image analysis, CNN

## Abstract

To safely select the proper therapy for ventricular fibrillation (VF), it is essential to distinguish it correctly from ventricular tachycardia (VT) and other rhythms. Provided that the required therapy is not the same, an erroneous detection might lead to serious injuries to the patient or even cause ventricular fibrillation (VF). The primary innovation of this study lies in employing a CNN to create new features. These features exhibit the capacity and precision to detect and classify cardiac arrhythmias, including VF and VT. The electrocardiographic (ECG) signals utilized for this assessment were sourced from the established MIT-BIH and AHA databases. The input data to be classified are time–frequency (tf) representation images, specifically, Pseudo Wigner–Ville (PWV). Previous to Pseudo Wigner–Ville (PWV) calculation, preprocessing for denoising, signal alignment, and segmentation is necessary. In order to check the validity of the method independently of the classifier, four different CNNs are used: InceptionV3, MobilNet, VGGNet and AlexNet. The classification results reveal the following values: for VF detection, there is a sensitivity (Sens) of 98.16%, a specificity (Spe) of 99.07%, and an accuracy (Acc) of 98.91%; for ventricular tachycardia (VT), the sensitivity is 90.45%, the specificity is 99.73%, and the accuracy is 99.09%; for normal sinus rhythms, sensitivity stands at 99.34%, specificity is 98.35%, and accuracy is 98.89%; finally, for other rhythms, the sensitivity is 96.98%, the specificity is 99.68%, and the accuracy is 99.11%. Furthermore, distinguishing between shockable (VF/VT) and non-shockable rhythms yielded a sensitivity of 99.23%, a specificity of 99.74%, and an accuracy of 99.61%. The results show that using tf representations as a form of image, combined in this case with a CNN classifier, raises the classification performance above the results in previous works. Considering that these results were achieved without the preselection of ECG episodes, it can be concluded that these features may be successfully introduced in Automated External Defibrillation (AED) and Implantable Cardioverter Defibrillation (ICD) therapies, also opening the door to their use in other ECG rhythm detection applications.

## 1. Introduction

Cardiac arrhythmia is prevalent in developed countries and represents a significant cause of mortality. Ventricular fibrillation (VF), even in its milder episodes, can lead to sudden cardiac death. As a result, the timely detection of ventricular arrhythmia is crucial to initiate appropriate therapeutic interventions and safeguard the patient’s life. While the causes of arrhythmia may vary, they all stem from disruptions in the heart’s cellular electrophysiology. Autopsy studies have consistently revealed that arrhythmogenic cardiac disorders are the primary underlying cause in cases of sudden cardiac death, with no evidence of pathological abnormalities in the heart. This underscores the fact that VF can trigger a rapid and irreversible degenerative process in the heart’s electrical system, leading to fatal consequences [1,2]. In order to restore normal cardiac rhythm during a ventricular fibrillation (VF) episode, the standard procedure involves the application of electrical defibrillation to the heart using an Automatic External Defibrillator (AED) [3]. AEDs are now readily available in various public locations, including airports, shopping centers, sports arenas, and more. This crucial process entails delivering a high-energy electrical shock externally, through the patient’s chest wall, with the aim of reestablishing a regular heart rhythm. Several studies [4,5,6] have demonstrated that the success of defibrillation is inversely correlated with the time elapsed between the onset of a ventricular fibrillation (VF) episode and the application of the electrical discharge. In other words, the longer the interval between the start of VF and the administration of the electrical shock, the lower the likelihood of a successful defibrillation. These findings underscore the critical importance of early intervention and prompt defibrillation in improving the chances of restoring a normal heart rhythm during VF episodes.

Detecting ventricular fibrillation (VF) automatically poses significant challenges due to its intrinsic characteristics, such as a lack of organization and irregularity, especially considering the existence of similar pathologies such as ventricular tachycardia (VT), where the required therapy is not the same as in VF. Specifically, some types of VT can be recovered by using drugs, and others require a low-energy synchronized electrical stimulation cardioversion. To successfully revert VF, an electrical shock must be administered, and the intensity of the shock (defibrillation level) depends on the stage of ventricular fibrillation. The early detection of VF enables the use of lower shock levels, facilitating faster restoration of the heart’s normal rhythms. However, it is of utmost importance to exercise caution, as administering an electrical shock to a patient not experiencing VF can lead to severe harm or even trigger VF. Ventricular tachycardia (VT) is one of the rhythms that can be particularly challenging to discern, underscoring the significance of accurate differentiation for making appropriate treatment decisions. Various detection algorithms have been developed utilizing diverse signal-processing techniques, including the Hilbert transform [7], Fourier transform [8], wavelet transform, and other signal processing methods [9,10], as well as time–frequency representations [11]. These techniques share a common characteristic: they integrate temporal and spectral information within the same representation. This fusion of information is particularly crucial when dealing with non-stationary processes like the electrocardiogram (ECG) signal, especially in the presence of irregular pathologies such as ventricular fibrillation (VF). By combining temporal and spectral information, these algorithms enable more effective detection and analysis of VF, enhancing our understanding and ability to diagnose and treat these irregular cardiac conditions.

The detection of ventricular fibrillation (VF) or ventricular tachycardia (VT) using electrocardiogram (ECG) data has been explored through numerous statistical methods. However, these manual approaches often struggle to extract features that effectively capture the intricate characteristics of ventricular arrhythmia. Consequently, machine learning techniques have emerged as successful alternatives for cardiac arrhythmia recognition. For instance, in [12], the wavelet method was implemented to identify ECG arrhythmias, specifically discerning three types of episodes: Normal, VT, and VF. In [13], a Support Vector Machine (SVM) with a Gaussian Kernel was employed to detect ventricular irregularities, utilizing morphological features. Furthermore, in [14], for the detection and classification of shockable arrhythmias (VF/VT) Random Forest (RF) decision trees were utilized in combination with Variational Mode Decomposition. In [15], the real-time identification of shockable episodes (VF/VT) was realized using fixed thresholds. Moreover, beyond these strategies, alternative studies have embraced a range of machine learning techniques for the identification and classification of ventricular arrhythmias. In [16], a C4.5 classifier was implemented. [17] employed a k-Nearest Neighbor (kNN) classifier while [18] utilized Bayesian decision methods. Additionally, [19] employed Decision Trees in conjunction with independent component analysis (ICA). By harnessing the power of machine learning, these approaches offer promising avenues to improve the accuracy and depth of ventricular arrhythmia detection. They enable the extraction of meaningful features and enhance the understanding and recognition of complex cardiac conditions. As a result, these advancements contribute to more effective diagnosis and treatment strategies for ventricular arrhythmia.

Applying traditional algorithms to leverage the information contained within the architecture of electrocardiogram ECG data poses a significant challenge, primarily due to the non-stationary nature of biomedical signals. Consequently, these conventional algorithms often exhibit limited performance when it comes to representing the intricate characteristics present in such complex data. In contrast, convolutional neural networks (CNNs) have garnered substantial interest in the scientific communities focused on image and speech classification. This heightened attention stems from the fact that the topology of CNNs closely resembles that of biological systems. As a result, CNNs offer a more suitable framework for capturing and analyzing the complex patterns inherent in ECG signals, allowing for improved performance in detecting and classifying cardiac conditions.

### 1.1. Related Work

Convolutional neural networks (CNNs) have found extensive application in various domains, including traffic sign detection [20], indoor object detection [21,22], and numerous other fields [23,24]. Recognizing faces poses a significant challenge and has garnered interest across different disciplines such as psychology, model identification, computer vision, and computer graphics. Consequently, the literature on face recognition is vast and diverse. In [25], the authors presented a long-distance face recognition method that addresses the variation in recognition rates caused by distance variations. They employed a CNN for face recognition and measured similarity using the Euclidean distance. This approach achieved outstanding performance at various distances, surpassing traditional face recognition methods. A hybrid system for face recognition was introduced by the authors in [26], combining a Logistic Regression Classifier (LRC) with a CNN. The CNN was trained to localize and identify faces in images, while the LRC classified the features learned by the convolutional network. Experimental results on the Yale face dataset [27] demonstrated improved classification accuracy and reduced processing time. In [28], a CNN-based face identification system with nine layers was proposed. The network consisted of three convolution layers, two pooling layers, two fully connected layers, and one Softmax layer. The proposed CNN was evaluated on the ORL face [29] and AR face datasets [30], achieving higher recognition rates compared to traditional machine learning and handcrafted feature methods for face identification. The implementation of a deep learning algorithm for face recognition was detailed in [31]. The algorithm was based on the OpenFace project, utilizing the FaceNet neural network architecture [32]. The results highlighted the effectiveness of the incremental learning algorithm in improving performance. An Active Face Recognition system (AcFR) was proposed in [33], which employed a CNN and mimicked human behavior in common face recognition scenarios. A pre-trained VGG-Face CNN was utilized to extract facial image features, followed by nearest-neighbor identity recognition for identification. Evaluation of the CMU PIE face dataset [34] demonstrated that the recognition stage of the AcFR system outperformed that of alternative systems. In [35], the authors introduced a novel face recognition system using a deep C2D–CNN model at the decision level.

### 1.2. Proposed Work

In this work, we propose a ventricular arrhythmia detection method, distinguishing VT and VF shockable rhythms, based on feeding a CNN with raw time–frequency data. It follows from the idea that the feature extraction from the matrix resulting from the time–frequency analysis using CNN allows better results to be obtained than those detectors using feature-selection strategies and reducing to a minimum the necessary signal preprocessing. In order to prove the validity of this method, a range of four CNN-based classifiers of different natures are used to evidence its independence of the classifier.

To achieve the objectives, this paper is structured as follows. Section 2 introduces the CNN algorithm, Section 4 describes the materials used and provides details on the processing applied to the ECG signal. Section 5, Section 6 and Section 8 present the results, discussions, and conclusions, respectively.

## 2. Deep Learning Algorithms

Deep learning models are neural networks that possess a deep structure inspired by the intricate workings of the human brain. By mimicking its processes, deep learning aims to address a wide range of learning problems. Particularly in the field of computer vision, deep learning techniques have achieved remarkable success. Currently, the main types of networks are multilayer perceptron, CNN, and recurrent neural network (RNN) [36]. As for other DL networks, such as fully convolutional networks (FCNs) they are typically used in tasks related to semantic segmentation [37].

### 2.1. Fundamental Concepts of Convolutional Neural Networks

In this section, we will introduce the widely recognized convolutional neural network (CNN) architecture and discuss the specific model utilized in this study.

**Figure 1:**
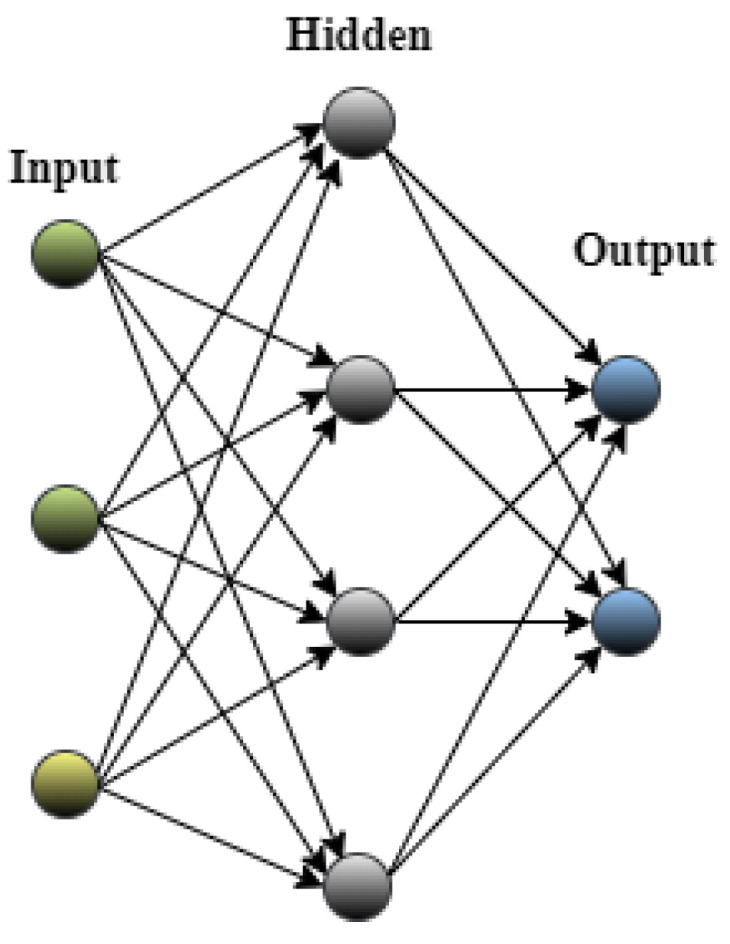
Artificial Neural Network (ANN).

As discussed earlier, CNNs are popular due to their improved performance in image recognition and classification. Architecture-wise, CNNs are simply feedforward Artificial Neural Networks (ANNs) [38,39], as illustrated in Figure 1. CNNs are characterized by their layered structure and employ filters, kernels, or neurons with learnable weights and biases. Each filter receives input, performs convolution operations, and may apply non-linear transformations [40]. A typical CNN architecture comprises the following components:

- The convolutional layer (CONV), which processes the received input data;
- The pooling layer (POOL), which allows compressing the information by reducing the size of the intermediate image (often by subsampling);
- The Fully Connected Layer (FCL) layer, which is a perceptron-type layer;
- The classification layer (Softmax), which predicts the class of the input image.

#### 2.1.1. Convolutional Layer

**Figure 2:**
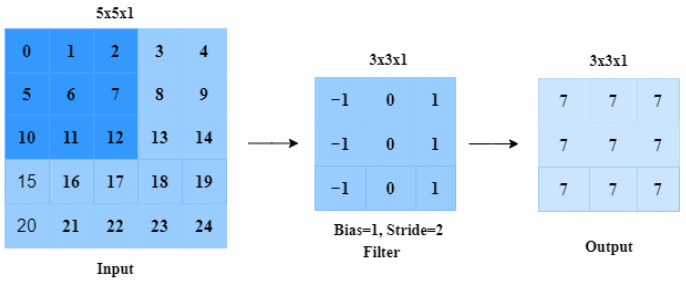
The process of a convolution operation.

The convolutional layer is a fundamental component of a Convolutional Network and plays a crucial role in the computational process. Its main function is to extract features from input data, particularly images. By applying convolution, the spatial correlation between pixels is preserved as the network learns image features using small squares of the input image. A set of learnable neurons convolve the input image, resulting in a feature map or activation map in the output image [36]. A kernel is placed in the top-left corner of the image. The process is repeated until all possible locations in the image are filtered, which is shown in Figure 2.

#### 2.1.2. Nonlinear Activation Function

**Figure 3:**
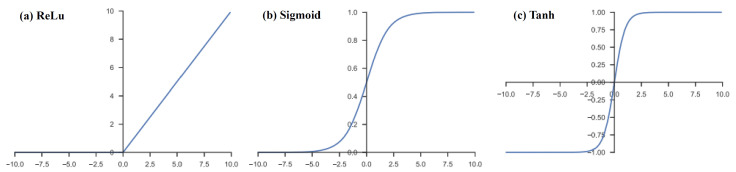
Activation functions commonly applied to neural networks: (**a**) rectified linear unit (ReLu), (**b**) Sigmoid, and (**c**) hyperbolic tangent (Tanh).

The results of a linear operation, such as convolution, undergo further processing through a nonlinear activation function. While smooth nonlinear functions like sigmoid or hyperbolic tangent (tanh) were previously utilized due to their resemblance to the behavior of biological neurons, the rectified linear unit (ReLU) has become the most popular choice for nonlinear activation functions. The ReLU function is defined as f(x)=max(0,x). Please refer to Figure 3 for a visual representation.

#### 2.1.3. Pooling Layer

**Figure 4:**
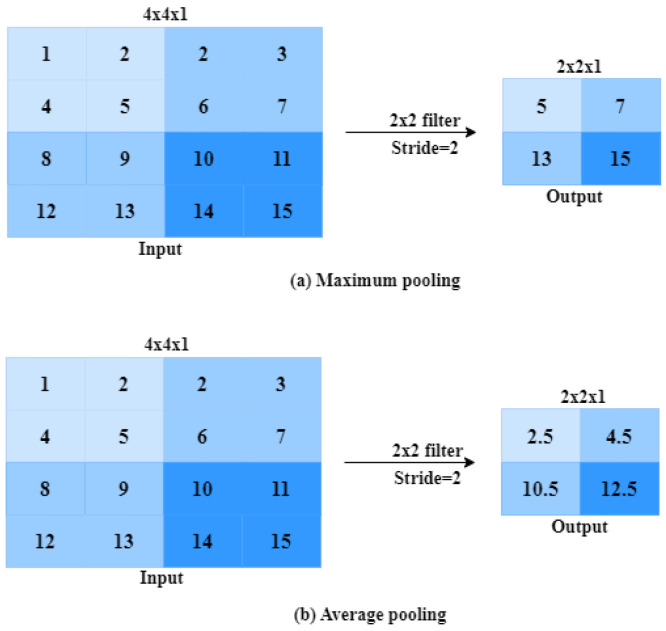
The process of pooling operation.

The pooling layer plays a crucial role in reducing the spatial size of the representation, thereby reducing the number of parameters and computational load in the network. Additionally, it helps to control overfitting. It is important to note that the pooling layer does not involve any learning process. Pooling units are generated using functions such as max-pooling, average pooling, or L2-norm pooling [36]. The process of the pooling operation is shown in Figure 4.

#### 2.1.4. Fully Connected Layer

The FCL serves as the final pooling layer, providing the extracted features to a classifier that uses the Softmax activation function [36]. The Softmax function ensures that the sum of the output probabilities from the Fully Connected Layer is 1. It achieves this by transforming a vector of arbitrary real-valued scores into a vector of values between zero and one that add up to one.

#### 2.1.5. Loss Function

A loss function, also known as a cost function, quantifies the agreement between the network’s output predictions obtained through forward propagation and the provided ground truth labels [41]. In multiclass classification tasks, the cross-entropy loss function is commonly used, while the mean squared error is typically employed for regression tasks involving continuous values. The selection of an appropriate loss function is a hyperparameter that depends on the specific task at hand and needs to be determined accordingly.

### 2.2. Optimization of Hyperparameters

Hyperparameters are parameters in a convolutional neural network (CNN) that are not learned during the training process but need to be specified beforehand. These hyperparameters significantly influence the network’s performance and can be adjusted to optimize the model’s accuracy and training efficiency. Some important hyperparameters in CNNs include the following.

- Number of layers [42]: A conventional CNN typically consists of multiple layers, including convolutional layers, activation layers (e.g., ReLU), pooling layers, and fully connected layers.
- Filter size (Kernel Size) [43]: The size of the filters used in the convolutional layers is an important parameter. Common filter sizes are 3 × 3, 5 × 5, and 7 × 7.
- Number of filters [44]: The number of filters in each convolutional layer determines the depth of the feature maps generated. More filters lead to more expressive power but also increase computation requirements.
- Stride [45]: The stride determines the step size at which the filter is moved across the input image. Common values are 1 and 2, with larger strides reducing the size of the output feature maps.
- Padding [45]: Padding can be used to preserve the spatial dimensions of the input when convolving with filters. Common padding values are ’same’ and ’valid’.
- Activation function [46]: Common activation functions include ReLU (rectified linear unit), leaky ReLU, and Sigmoid. ReLU is widely used due to its simplicity and effectiveness.
- Pooling [47]: Pooling layers downsample the feature maps reduces the spatial dimensions. Common pooling types are Max pooling and average pooling, typically with a pool size of 2×2.
- Fully connected layers [48]: The number of neurons in the fully connected layers can vary based on the complexity of the task. The output layer size depends on the number of classes in the classification task.
- Dropout [49]: Dropout is a regularization technique that randomly sets a fraction of neurons to zero during training, preventing overfitting. Common dropout rates are between 0.2 and 0.5.
- Batch size [50]: The number of samples used in each iteration during training. Smaller batch sizes are computationally more expensive but can lead to better convergence.
- Number of epochs [51]: This is the number of times the entire training dataset is passed through the network during training.
- Learning rate [52]: The learning rate controls the step size during optimization. A small learning rate leads to slow convergence, while a large learning rate can cause instability.
- Optimizer: Common optimizers used in CNNs include Stochastic Gradient Descent (SGD) [53], Adam, and RMSprop.

The choice of these parameters depends on the specific problem, dataset, and available computing resources. Often, hyperparameter tuning and experimentation are required to find the best parameter settings for a given CNN architecture and task.

### 2.3. CNN Architectures

In this study, four different CNN architectures were employed: AlexNet, VGGNet, InceptionV3, and MobileNet.

#### 2.3.1. AlexNet

AlexNet is a deep CNN architecture capable of classifying over 1000 different classes. It consists of five convolutional layers (CLs) with three pooling layers, two fully connected layers (FLCs), and a Softmax layer. AlexNet utilizes a total of 650 k neurons and 60 million parameters. The input image for AlexNet needs to have dimensions of 227×227×3. The first CL takes the input image and applies 96 kernels of size 11×11×3 with a stride of four pixels, producing the output for the second layer [54].

#### 2.3.2. VGGNet

VGGNet, short for the visual geometry group network, is a deep neural network known for its multilayered architecture. It is based on the CNN model and has been widely applied to the ImageNet dataset. VGG-19, in particular, is known for its simplicity and utilization of 3×3 convolutional layers, which contribute to its increased depth. Max pooling layers are used to reduce the volume size in VGG-19, and it includes two fully connected (FC) layers with 4096 neurons [55].

#### 2.3.3. Inception V3

The Inception V3 is a deep learning model based on convolutional neural networks, which is used in image analysis and object detection. Inception V3 is a superior version of the basic model Inception V1, which was introduced by Szegedy and others in 2014 [56].

#### 2.3.4. MobileNet

The MobileNet model is specifically designed for efficiency and optimized for running on embedded or mobile devices. Its key layer is the depthwise separable convolution, which helps reduce the number of features. MobileNet v2, released in April 2017, introduced bottleneck layers and shortcut connections as updates from the previous version [57].

## 3. Time–Frequency Representation

**Figure 5:**
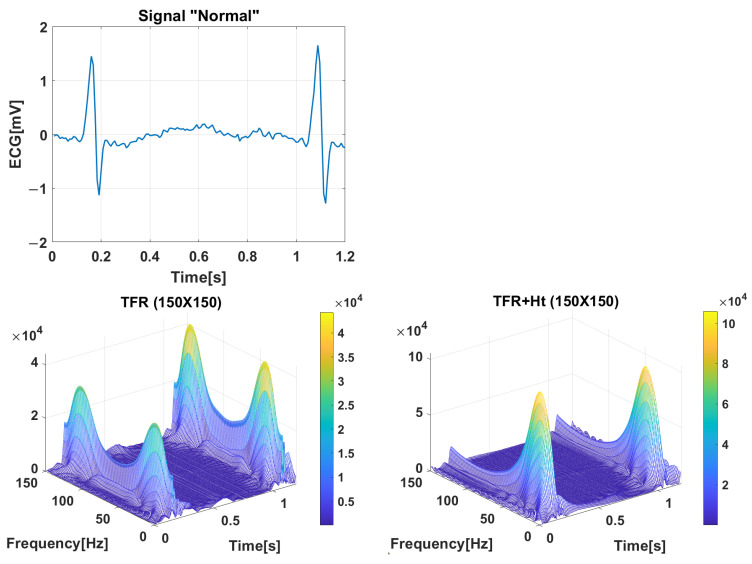
PWV distribution of the ECG Normal signal directly processed without the Hilbert transform. PWV distribution of the Normal analytic signal using the Hilbert transform.

The Wigner–Ville Distribution (WV) is one of the most commonly used representations for time–frequency analysis. It is applied to the ECG time window without applying the Hilbert transform before performing the time–frequency decomposition. Figure 5 shows the symmetry of the diagram due to the presence of both positive and negative frequencies. In the second case, the analytic signal is first calculated using the Hilbert transform, and then each matrix is processed using the WV based on the obtained analytic signal.

Compared to the PWV, the artifacts and interferences introduced by the WV have been reduced, allowing for clearer spectral visualization [58], so the Pseudo Wigner–Ville (PWV) variant was finally used. This variant reduces these terms using a smoothing kernel h(t). The mathematical description of PWV is defined as shown in the equation below.

(1) $$PWV_{x}=\int_{-\infty }^{+\infty }h\left(\tau \right)S\left(t+\frac{\tau }{2}\right)S^{*}\left(t+\frac{\tau }{2}\right)e^{-j2\nu \pi \tau }d\tau$$

where S(t) is the analyzed signal, τ is the time lag, t is the time instant, and h is the frequency smoothing window. In order to reduce interference, PWV uses the analytic signal to replace the original signal filtering out and thus the negative frequency. The analytic signal S(t) corresponding to the original x(t) signal is given by Equation (2).

(2) S[x(t)]=x(t)+jH[x(t)]

where H[x(t)] is the Hilbert transform of x(t), as shown in Equation (3).

(3) $$H\left[x\left(t\right)\right]=\frac{1}{\pi }\int_{-\infty }^{+\infty }\frac{x(\tau )}{t-\tau }d\tau$$

## 4. Material and Methods

**Figure 6:**
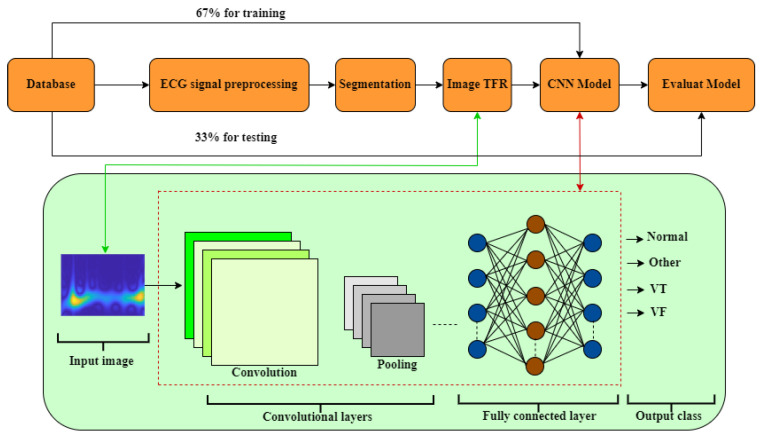
A comprehensive diagram outlines the series of processing steps applied in the detection of ventricular fibrillation.

Figure 6 shows the general scheme of the followed methodology, from the reading of the records of the database to the results obtained by the classifier.

The developed methodology is composed of four fundamental phases.

- First phase: The dataset used is described.
- Second phase: The ECG data undergoes filtering to reduce baseline interference. Once filtered, the Window Reference Mark (WRM) of the ECG signal is obtained. Each WRM indicates the start of a time window (tw) within the ECG signal.
- Third phase: Information extraction is performed by applying the Hilbert transform (Ht) to each window tw obtained in the first phase. Subsequently, the TFR matrix is computed using the Pseudo Wigner–Ville method, resulting in the Time–Frequency Representation Image (TFRI).
- Fourth phase: The TFRI matrices obtained in the previous step are used as input for a deep learning CNN (CNN1, CNN2, InceptionV3, MobilNet, VGGNet, and AlexNet), as detailed in Section 2.3 and Section 4.4.1. The success of ventricular fibrillation (VF) detection relies on signal processing techniques and the structure of the classifiers employed. To achieve optimal performance, it is necessary to adjust the CNN parameters to better adapt to the data.

### 4.1. Materials

The ECG records used in this study were sourced from the MIT-BIH Malignant Ventricular Fibrillation [59] and AHA (2000 series) [60] standard databases. Without preselecting ECG episodes, the analysis was conducted to simulate the use of an AED. A total of 24 patients were included in the analysis, consisting of 22 records from the MIT-BIH database and two additional records from the AHA database. Each record contained half-hour annotated ECG recordings of continuous ECG. The inclusion of AHA records was intended to increase the number of ventricular tachycardia (VT) episodes and improve the balance of recorded time between ventricular tachycardia (VT) and ventricular fibrillation (VF) episodes. The study defined four groups (classes) of rhythms: normal sinus rhythm (Normal), ventricular tachycardia (VT), ventricular fibrillation including flutter episodes (VF), and other rhythms (non-ventricular arrhythmia, noise, etc.), labeled as Other (Other).

### 4.2. Electrocardiographic Signal Preprocessing

#### 4.2.1. Denoising

**Figure 7:**
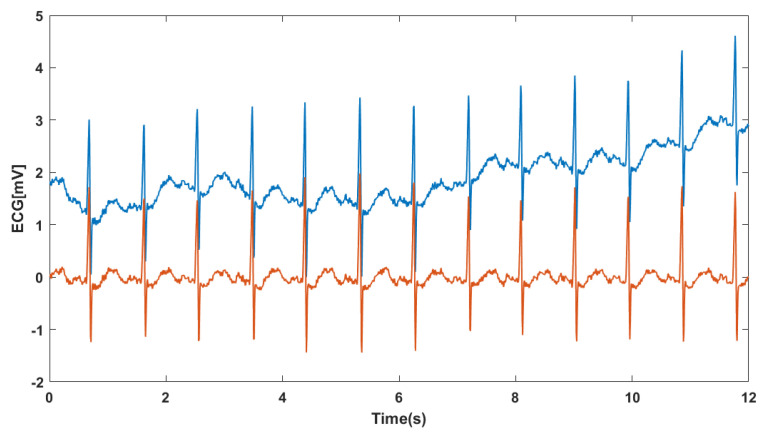
IIR bandpass filter applied to a Normal-type ECG. The original temporal signal is plotted in blue, and the filtered output signal is shown in red. The frequency response of the filter is displayed below.

The purpose of this preprocessing stage is to eliminate various types of noise present in the ECG signal, such as baseline oscillation and interferences like power line interference and electromyogram (EMG). Baseline oscillations typically have a frequency range below 1 Hz, power line interference occurs at 50 or 60 Hz, and the EMG exhibits a wide bandwidth with low amplitude when the patient is at rest and with a low energy below 45 Hz. To address these issues, the ECG signal is first resampled to 125 Hz. Then, an 8th-order IIR bandpass filter with a Butterworth response is applied, with a passband ranging from 1 Hz to 45 Hz. This effectively removes the baseline oscillation below 1 Hz, power line interference, and EMG activity above 45 Hz [61,62], as illustrated in Figure 7.

#### 4.2.2. Segmentation

The next step involves obtaining a Window Reference Mark (WRM) to indicate the beginning of the ECG time window, denoted as tw. According to [58], a normal heart rate range is considered to be between 50 and 120 beats per minute (bpm). Therefore, the minimum distance ($WRM_{min}$) and maximum distance ($WRM_{max}$) between two consecutive WRMs are set to 0.5 s and 1.2 s, respectively. These values were utilized in our analysis. The calculation of WRM reference marks was performed using a pre-existing algorithm, where $N_{LMC}$ represents the number of local maxima LM marks present in the signal. From each generated WRM reference mark, a time window $tw_{j}$ of 1.2 s in length (150 samples) was created, starting at the corresponding WRM mark $WRM_{j}$, as shown in Equation (4).

(4) $$\begin{array}{c} tw_{j}=\left[WRM_{j},WRM_{j}+1.2\;s\right];\;j=1,\cdots ,N_{LMC} \end{array}$$

### 4.3. Extraction of Image from TFR

Once the data matrix is obtained from the Time–Frequency Representation (TFR) combined with the Hilbert transform (Ht) for each tw window, this data matrix TFR is converted into an image TFRI (Lf × Lt) with a size of Lf × Lt pixels, where Lf = 45 and Lt = 150. This image is then directly input into the CNN. This approach ensures that all temporal and spectral information from the ECG signal is preserved in the data matrix, providing the classifier with comprehensive data information. It is important to note that there is no feature extraction performed on the TFRI, as it already contains the temporal and spectral information of the ECG signal.

**Figure 8:**
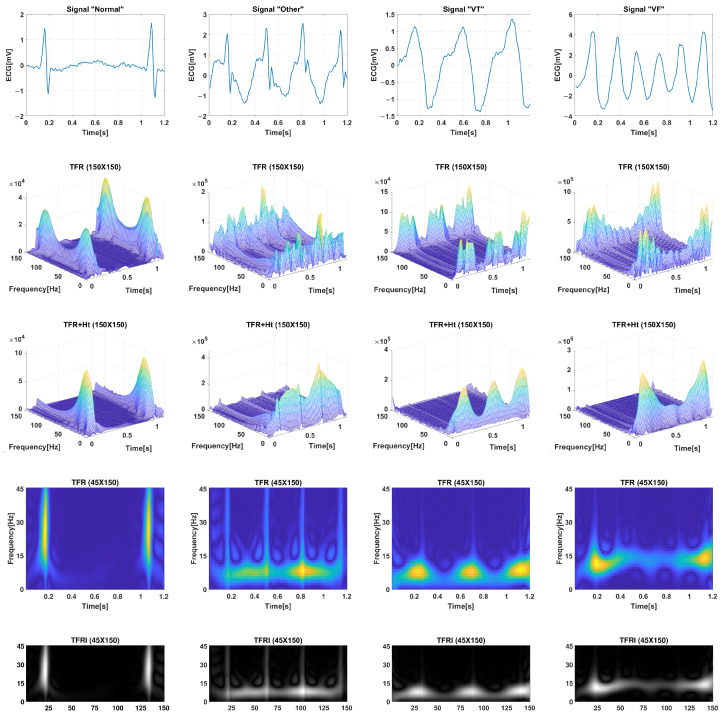
In the illustration, the columns, from top to bottom, represent the original ECG time signal window, TFR (150×150), TFR + Ht (150×150), TFR + Ht (45×150), and TRFI (45×150), respectively. From left to right, they correspond to the classes Normal, Other, VT, and VF, respectively.

Figure 8 illustrates examples of the time–frequency representations (TFR) using the Pseudo Wigner–Ville (PWV) transform for signals belonging to the Normal, Other, VT, and VF classes. The intensity distributions clearly exhibit distinct patterns for each class. In the case of a Normal signal, the intensity is localized in time, primarily due to the QRS complex, and it covers a wide range of frequencies. On the other hand, VF signals exhibit irregular intensity distributions along both the time and frequency axes without a specific pattern.

### 4.4. Model Training and Evaluation

#### 4.4.1. Model Architecture

The architectures of the proposed CNN model are summarized in Table 1.

- In the CNN1 method, 2 fully connected layers utilize the output from the TFR and predict the class of the image based on the vector calculated in previous stages.
- In the CNN2 method, the network consists of 6 layers, including 2 convolution layers, 2 max-pooling layers, and 2 fully connected layers. Each convolution layer (layers 1 and 2) applies convolution with its respective kernel size (layers 3 and 4). Following each convolution layer, a max-pooling operation is performed on the generated feature maps. The purpose of max-pooling is to reduce the dimensionality of the feature maps, aiding in the extraction of essential features.

#### 4.4.2. Training the Convolutional Neural Network Model

**Figure 9:**
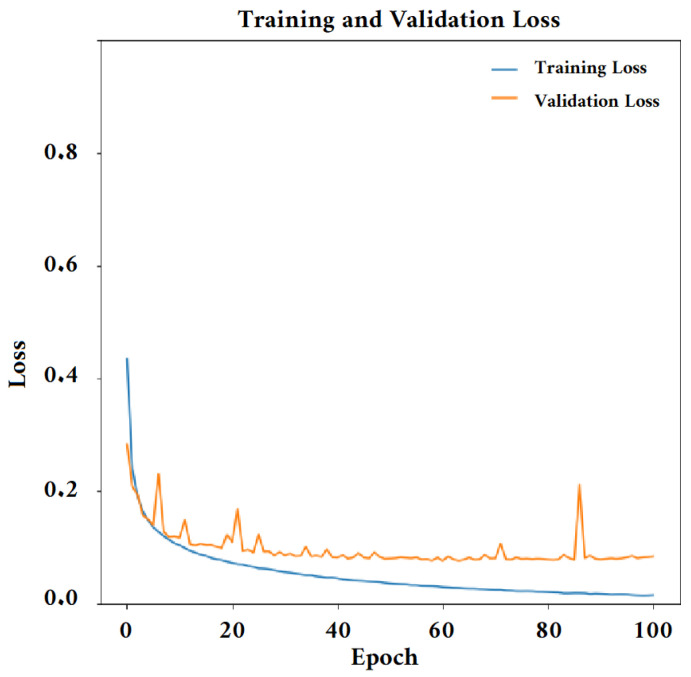
Loss function diagram. The figure shows the function image of the model training CNN2; the train loss is 0.02, and the val loss is 0.1.

**Figure 10:**
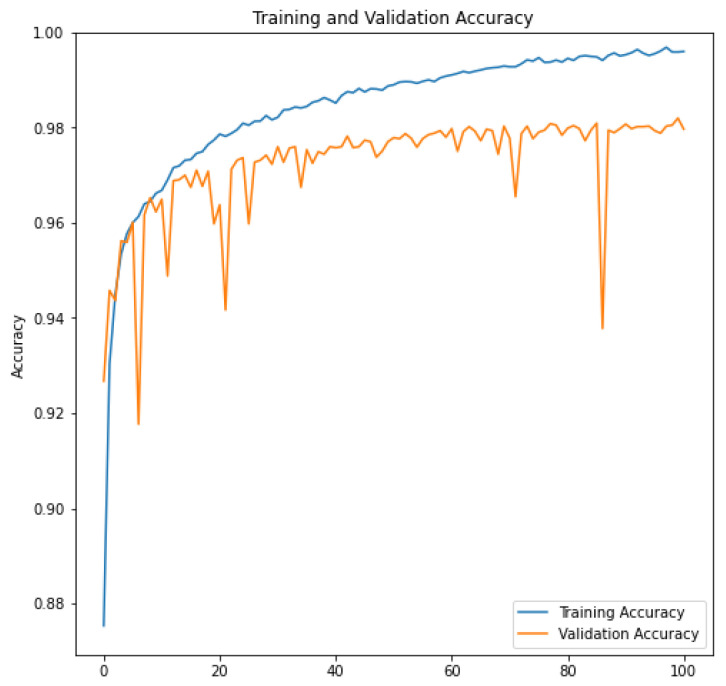
Accuracy function. The figure shows the function image of model training Ht_TFR_CNN2; the train accuracy is 100%, and the val accuracy is 98%.

Unlike other research studies, which utilized optimization techniques to select layers in complex CNN architectures and employed different hyperparameters for training, in our case, we have taken a different approach. We began with a basic CNN structure and conducted a series of systematic tests where we progressively added and adjusted layers. Throughout this process, we maintained consistent hyperparameters for training. We evaluated the impact of these layers on performance using a validation dataset. This unique methodology has enabled us to identify the specific layers that have a notable positive impact on the network’s performance for the particular task we are addressing. The Adam optimizer was employed for training the model, and the categorical cross-entropy loss function was utilized for this purpose. The model was trained for 100 epochs. The training and validation results are depicted in Figure 9 and Figure 10. We can see that the error is close to 0 and the accuracy value is very high in both the training and evaluation sets. This indicates that training with 100 epochs is sufficient to have a well-trained model.

Cross-validation is essential for selecting optimal parameters in machine learning and deep learning. Various traditional cross-validation methods are available, such as leave-one-out cross-validation and k-fold cross-validation [63]. In this study, we followed a specific approach. We randomly chose 67% of the data for each class for training, leaving 33% for testing. The CNN model was trained on the training data, and we evaluated its classification performance on the test data employing metrics like sensitivity, specificity, a, and F-Score. We repeated this process five times with different random selections and averaged the results to assess the overall classifier performance.

### 4.5. Performance Metrics for Classification

The performance of different networks on the testing dataset was evaluated after the completion of the training phase. The evaluation was based on four performance metrics: accuracy, sensitivity, specificity, and F-Score. The following equations were used for calculation [64,65]:

(5) $$\begin{array}{c} Accuracy\left(\%\right)=\frac{\left(TP+TN\right)}{\left(TP+FP+TN+FN\right)}\times 100 \end{array}$$

(6) $$\begin{array}{c} Sensitivity\left(\%\right)=\frac{\left(TP\right)}{\left(TP+FN\right)}\times 100 \end{array}$$

(7) $$\begin{array}{c} Specificity\left(\%\right)=\frac{\left(TN\right)}{\left(TN+FP\right)}\times 100 \end{array}$$

(8) $$\begin{array}{c} FScore\left(\%\right)=\frac{\left(2\times TP\right)}{\left(2\times TP+FP+FN\right)}\times 100 \end{array}$$

In the classification of Normal, Other, VT, and VF patients, the terms true-positive (TP), true-negative (TN), false-positive (FP), and false-negative (FN) were used.

## 5. Results

The preprocessing stage involved denoising and reducing baseline variation by applying an eighth-order Butterworth IIR bandpass filter with a frequency range of 1 Hz to 45 Hz. Window reference marks (WRMs) were calculated to indicate the beginning and end of the 1.2 s time window for each temporal signal. As previously mentioned, the experiments in this study utilized signals extracted from the MIT-BIH and AHA standard databases, categorized into four distinct groups: VF, VT, Normal, and Other. The initial preprocessing step encompassed denoising and baseline variation reduction through the utilization of an eighth-order Butterworth IIR bandpass filter with a frequency range spanning from 1 Hz to 45 Hz. Furthermore, window reference marks (WRMs) were generated to delineate the temporal boundaries of the 1.2 s time window (tw) for each signal.

We have proposed three different techniques to extract the image feeding the classifier: TFR_CNN1, Ht_TFR_CNN1, and Ht_TFR_CNN2.

- In the TFR_CNN1 approach, we initially transformed each tw into a time–frequency Representation Image (TFRI) utilizing the Pseudo Wigner–Ville transform, without using the Hilbert transform (Ht). The resulting image was then converted into a feature vector, which served as input for the Fully Connected Layer (FCL) of the classifier.
- In the Ht_TFR_CNN1 method, information extraction involved applying the Hilbert transform to each window’s tw obtained in the first phase, followed by the assessment of the Time–Frequency Representation (TFR) matrix using the Pseudo Wigner–Ville transform. The resulting TFR matrix was used to generate the TFRI, which was then used as input for the FCL.
- In the Ht_TFR_CNN2 method, the parameters were extracted using CNN2 by combining the Hilbert transform (Ht) and the TFRI. The extracted vectors were then used as input for the FCL.

In the TFR_CNN1, Ht_TFR_CNN1, and Ht_TFR_CNN2 methods, after receiving a vector at the input, the FCL applies a linear combination and an activation function successively to classify the input image. The output of the FCL is a vector of a size corresponding to the number of classes, where each component represents the probability of the input image belonging to a specific class.

**Figure 11:**
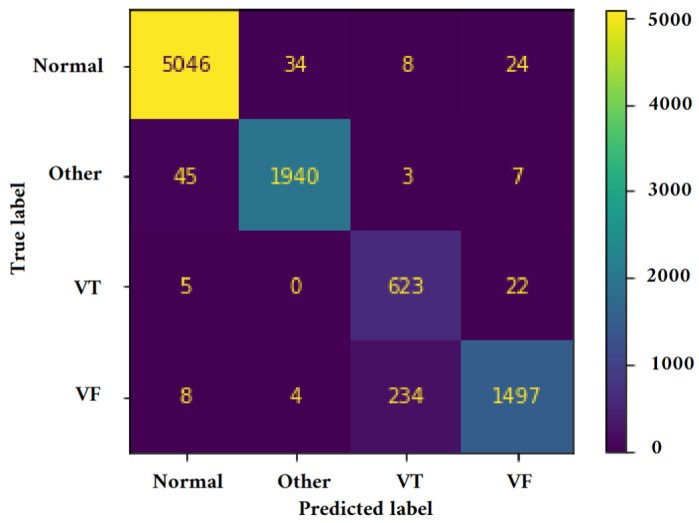
Confusion matrix for classifying Normal, Other, VT, and VF classes utilizing the TFR_CNN1 technique (Epochs = 50).

**Figure 12:**
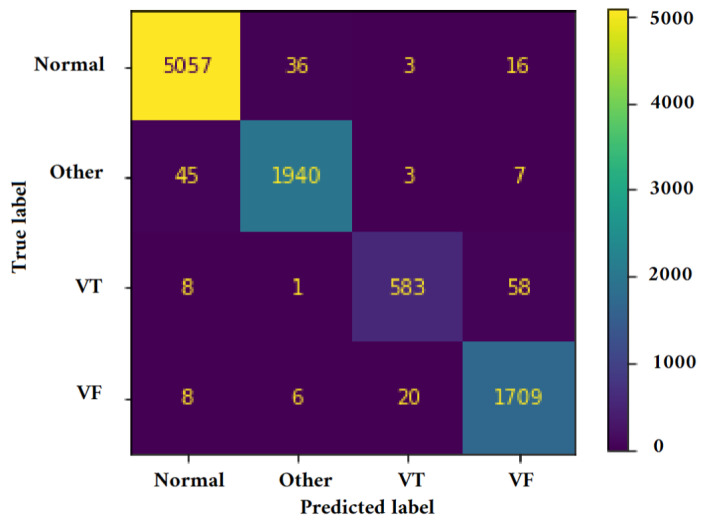
Confusion matrix for classifying Normal, Other, VT, and VF classes utilizing the Ht_TFR_CNN1 technique (Epochs = 50).

**Figure 13:**
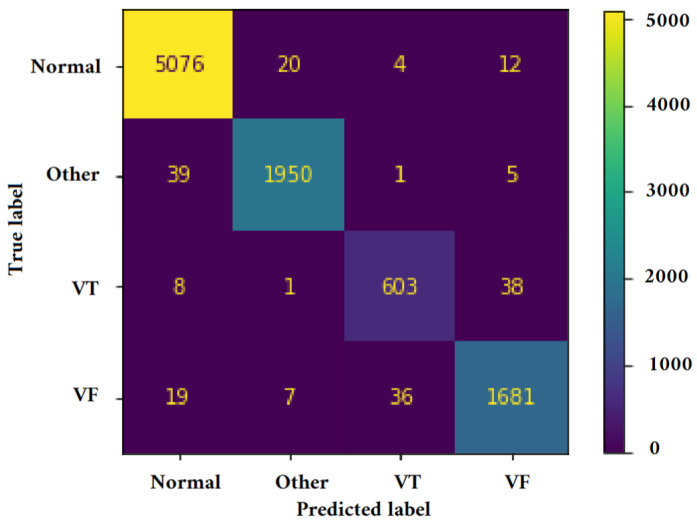
Confusion matrix for classifying Normal, Other, VT, and VF classes utilizing the Ht_TFR_CNN1 technique (Epochs = 100).

**Figure 14:**
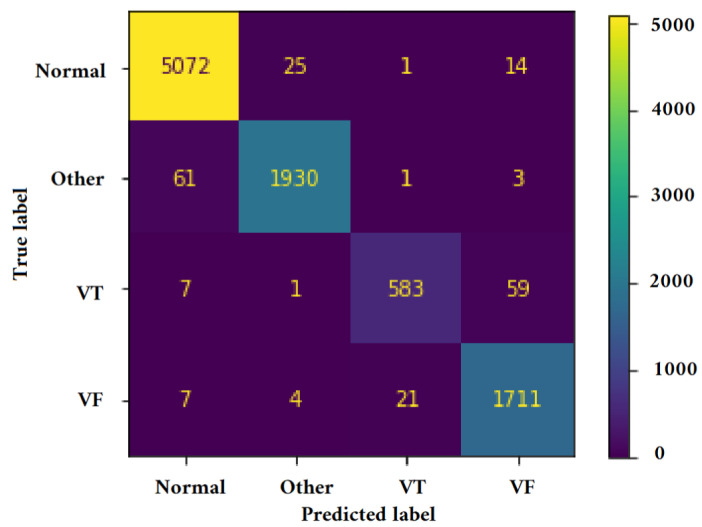
Confusion matrix for classifying Normal, Other, VT, and VF classes utilizing the Ht_TFR_CNN2 method (Epochs = 100).

Figure 11, Figure 12, Figure 13 and Figure 14 illustrate the confusion matrix for one of the iterations. Table 2, Table 3, Table 4 and Table 5 present the averaged performance values acquired from the reiterated random validation employed in this study. When the TFR_CNN1 algorithm was used (epochs = 50), the results showed a sensitivity of 85.88%, an overall specificity of 99.30%, an overall accuracy of 96.82%, and an overall F-Score of 92.10% for VF, and a sensitivity of 95.84%, an overall specificity of 97.19%, an overall accuracy of 97.09%, and an overall F-Score of 96.52% for VT. It can be concluded that achieving high classification results using the TFR_CNN1 strategy is challenging, primarily due to the significant similarity between VF and VT signals. This necessitates the exploration of alternative approaches to address the class discrimination problem, leading to the utilization of Ht with RTF. The results obtained using the Ht_TFR_CNN1 algorithm (epochs = 50) for VF detection showed a sensitivity of 98.04%, an overall specificity of 98.94%, an overall accuracy of 98.77%, and an overall F-Score of 98.48%, while for VT, a sensitivity of 89.70%, an overall specificity of 99.70%, an overall accuracy of 99.00%, and an overall F-Score of 94.43% were obtained. When employing the Ht_TFR_CNN1 algorithm (epochs = 100) for VF detection, a sensitivity of 96.44%, an overall specificity of 99.28%, an overall accuracy of 98.75%, and an overall F-Score of 97.83% were achieved. For VT, the results included a sensitivity of 92.70%, an overall specificity of 99.53%, an overall accuracy of 99.06%, and an overall F-Score of 95.99%.

In the analysis of VF and VT detection using the Ht_TFR_CNN1 (epochs = 50) and Ht_TFR_CNN1 (epochs = 100) methods, it can be observed that both sensitivity and overall specificity fall within the range of 89.70% to 99.70%. These results are superior to those obtained without utilizing Ht, indicating their considerable acceptability, and consequently, they were chosen for subsequent tests. Regarding the Ht_TFR_CNN1 (epochs = 100) method, the results are better than those obtained using Ht_TFR_CNN1 (epochs = 50), indicating a better learning capability of the training dataset.

### Analysis Based on Different CNN Algorithms

**Figure 15:**
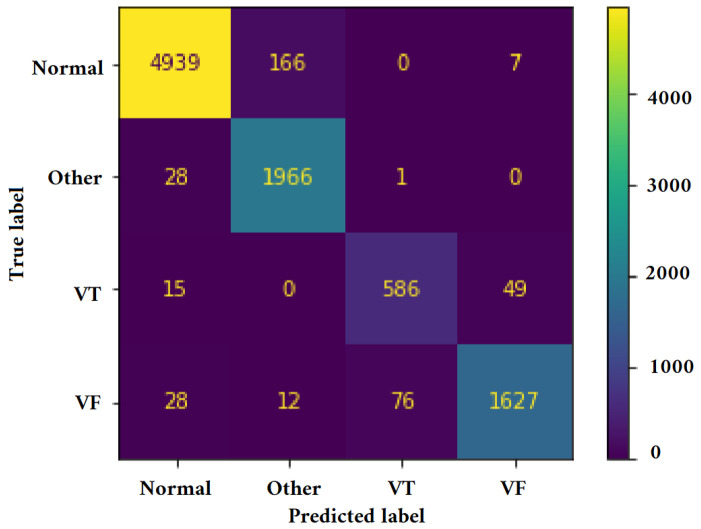
Confusion matrix for classifying Normal, Other, VT, and VF classes utilizing the VGGNet method (Epochs = 6).

**Figure 16:**
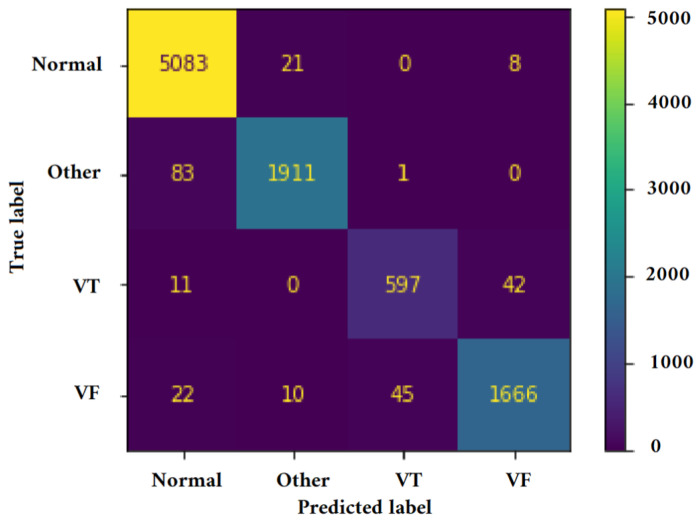
Confusion matrix for classifying Normal, Other, VT, and VF classes utilizing the Alexnet method (Epochs = 6).

**Figure 17:**
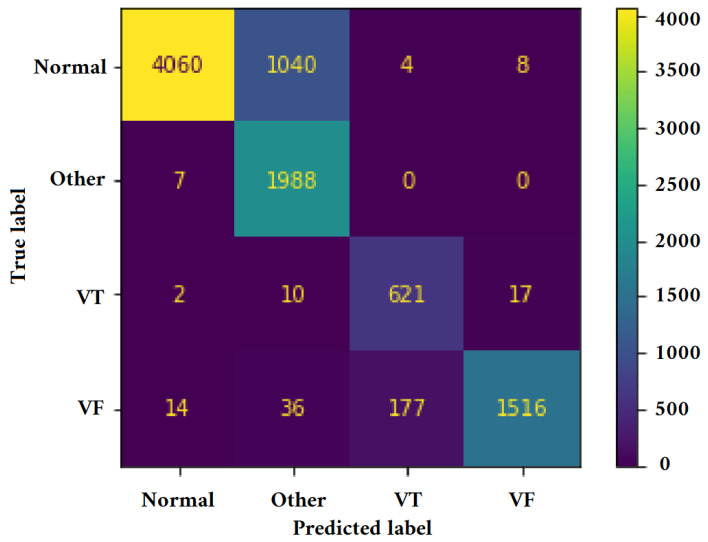
Confusion matrix for classifying Normal, Other, VT, and VF classes utilizing the Mobilnet method (Epochs = 6).

**Figure 18:**
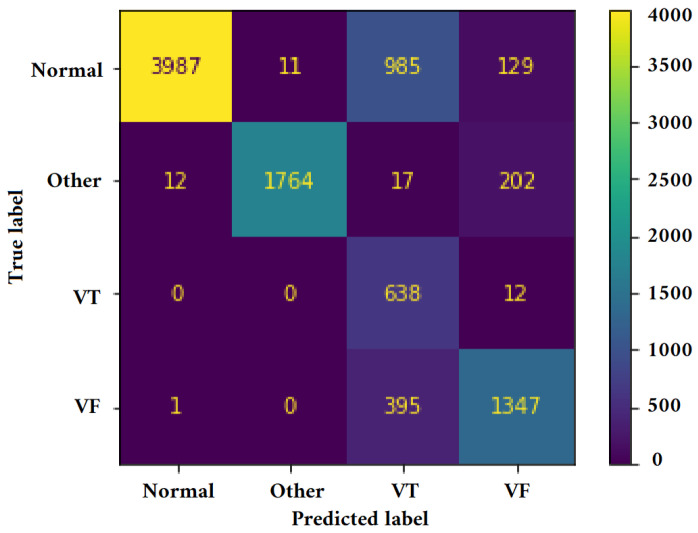
Confusion matrix for classifying Normal, Other, VT, and VF classes utilizing the InceptionV3 method (Epochs = 6).

**Figure 19:**
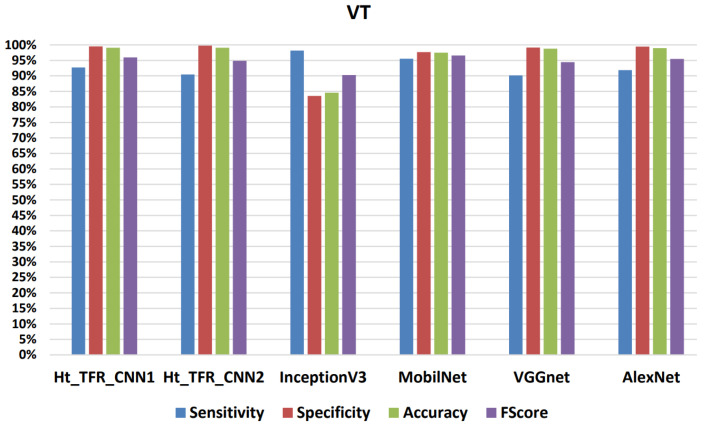
Results achieved for the classification of the VT class during testing.

**Figure 20:**
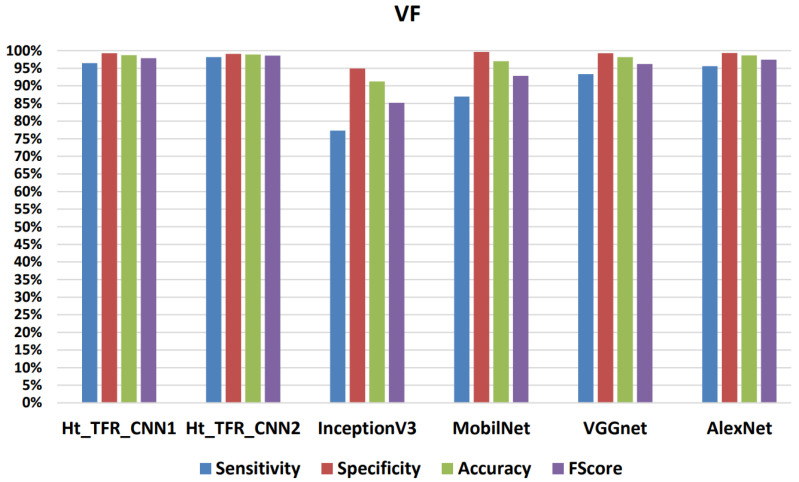
Results achieved for the classification of the VF class during testing.

Figure 15, Figure 16, Figure 17 and Figure 18 present the confusion matrix derived from one of the five iterations of testing data. Additionally, we enhance the understanding of these findings by presenting Table 6, Table 7, Table 8 and Table 9, and Figure 19 and Figure 20, which summarize the results obtained from comparing the sensitivity, specificity, accuracy, and F-Score values achieved for the respective four classes.

When comparing the classifiers VGGNet and AlexNet with MobilNet and InceptionV3, it is evident that the former two yield better results, demonstrating a higher learning capability with the dataset. Analyzing the values in Table 8 and Table 9, when using the VGGNet classifier for VT, a sensitivity of 90.15%, overall specificity of 99.15%, overall accuracy of 98.77%, and overall F-Score of 94.43% were obtained. For VF, a sensitivity of 93.34%, overall specificity of 99.25%, overall accuracy of 98.14%, and overall F-Score of 96.20% were achieved. Similarly, using the AlexNet classifier for VT resulted in a sensitivity of 91.84%, overall specificity of 99.47%, overall accuracy of 98.94%, and overall F-Score of 95.50%. For VF, a sensitivity of 95.58%, an overall specificity of 99.34%, an overall accuracy of 98.64%, and an overall F-Score of 97.42% were obtained.

On the other hand, the classifiers Ht_TFR_CNN1 and Ht_TFR_CNN2 exhibit similar behavior for the classes Normal and Others.

For the Normal class, they showed a sensitivity of 99.29%, 99.34%; an overall specificity of 98.62%, 98.35%; an overall accuracy of 98.91%, 98.89%; and an overall F-Score of 98.95%, 98.84%, respectively. For the Others class, they displayed a sensitivity of 97.74%, 96.98%; an overall specificity of 99.62%, 99.68%; an overall accuracy of 99.22%, 99.11%; and an overall F-Score of 98.67%, 98.31%, respectively. However, the InceptionV3 classifier has a higher sensitivity of 98.15% for VT and a lower sensitivity of 77.28% for VF compared to the Ht_TFR_CNN2 classifier, which exhibits a lower sensitivity of 90.45% for VT and a higher sensitivity of 98.16% for VF. Comparing the results provided by the different algorithms, there is a significant variation in the sensitivity results for VF and sensitivity results for VT, primarily due to the morphological similarities between the VT class and the VF class.

## 6. Discussion

The identification of ventricular arrhythmias generally involves a procedure for extracting and selecting relevant features. In this study, we proposed using the Ht_TFR_CNNi method with (i=1,2) to extract features that capture information about the shape of the ECG signal. This combined method of Ht and TFR with CNN aims to condense the relevant information about the data’s shape, enabling effective detection and discrimination of shockable VF and VT rhythms, even in the presence of noise and complex signals. The obtained results shown in Table 2, Table 3, Table 4, Table 5, Table 6, Table 7, Table 8 and Table 9 demonstrate the use of the CNN classifier with input features obtained from two methods, namely Ht_TFR_CNN1 and TFR_CNN1. The results indicate that the Ht_TFR_CNN1 and Ht_TFR_CNN2 features yield better performance, which is why we compare the Ht_TFR_CNN2 method with other works in the literature. While we employed the CNN classifier to highlight the enhanced classification outcomes compared to prior studies, the investigation of alternative classifiers remains an ongoing avenue that could potentially yield further improvements.

As shown in Table 10, the proposed Ht_TFR_CNN2 method achieves an average accuracy of 98.91% for multi-class discrimination, effectively distinguishing Normal, Other, and VT, VF types of ventricular arrhythmia. Additionally, Table 11 presents a two-class classification approach, demonstrating that the Ht_TFR_CNN2 method achieves an accuracy of 99.61% in discriminating shockable (VT or VF) and non-shockable rhythms. These results indicate that the Ht_TFR_CNN2 method delivers high classification performance. However, we also provide a comparison with other works in the literature, although it is challenging due to differences in the source signals used and the specific discrimination tasks performed. To compare with works focusing on VF discrimination, our work achieved high classification performance [58] by feeding the complete time–frequency image as input to different classifiers (e.g., Sen = 92.8% and Spe = 97.0% for VF, and Sen = 91.8% and Spe = 98.7% for VT, using an Artificial Neural Network Classifier, ANNC), Arafat et al. [66] achieved (Sens = 80.97%, Spe = 98.51%) for classifying VF episodes utilizing an improved version of the Threshold Crossing Interval (TCI) algorithm. Roopaei et al. [67] obtained an Acc = 88.60% utilizing chaotic-based reconstructed phase space features. In [68] attained Sens=91.9% and Spe =97.1% in detecting VF episodes employing SVM and specific feature-selection classifiers. Li and Rajagopalan [69] utilized a genetic algorithm and obtained Sens = 98.40%, Spe = 98.00%, and Acc = 96.30% in discriminating VF episodes. Ibtehaz et al. [70] achieved the highest results in this group, employing SVM and Empirical Mode Decomposition (EMD) classifiers (Sens = 99.99%, Spe = 98.40%, and Acc = 99.19%) for VF and non-VF classification. Acharya et al. [71] detected and classified ventricular arrhythmias employing a CNN neural network, achieving Sen = 56.44%, Spe = 98.19%, and Acc = 97.88% for VF. Xia et al. [72] obtained high performance values (Sen = 98.15% and Spe = 96.01% for VF, and Sen = 96.01% and Spe = 98.15% for VT) using Lempel–Ziv and Empirical Mode Decomposition (EMD) with selected clean episodes of VT and VF. Mjahad et al. [73] achieved an accuracy, sensitivity, and specificity values of 98.68%, 92.72%, and 99.53%, respectively employing TDA. Kaur and Singh [74] used Empirical Mode Decomposition (EMD) and approximate entropy with selected VF and VT episodes from the MIT-BIH database, achieving moderate classification performance (Sen = 90.47%, Spe = 91.66%, and Acc = 91.17%). In [75], the authors proposed a fuzzy similarity-based approximate entropy approach and obtained high performance ratios (Sen = 97.98% and Spe = 97.03% for VF, and Sen = 97.03% and Spe = 97.98% for VT). However, a fair comparison must consider that Xie’s work involved the preselection of clean episodes of VF and VT. Despite the preselection of ECG episodes in some works, the results of the Ht_TFR_CNN method in this study outperform the other works in the literature aiming to discriminate between VF and VT rhythms. 

Table 11 presents a comparison focusing on detecting VT/VF episodes, specifically shockable and non-shockable rhythms. This set of works primarily targets the implementation on external defibrillators (AEDs) and implantable cardioverter defibrillators (ICDs), distinguishing between shockable and non-shockable rhythms (considering both VT and VF as shockable). Mjahad et al. [73] utilized TDA and obtained Sens = 99.03%, Spe = 99.67%, and Acc = 99.51% in discriminating VF episodes. Acharya et al. [76] proposed an eleven-layer convolutional neural network (CNN) for shockable and non-shockable arrhythmia classification, obtaining Sen = 95.32%, Spe = 91.04%, and Acc = 93.20%. Tripathy et al. [14] proposed Variational Mode Decomposition (VMD) and the Random Forest (RF) classifier, achieving Sen = 96.54%, Spe = 97.97%, and Acc = 97.23%. Buscema et al. [77] obtained Acc = 99.72% utilizing RNN. Kumar et al. [78] obtained Acc =98.80%, Sen = 98.60%, and Spe =98.90% employing CNN and IENN. Alonso-Atienza et al. [68] also obtained accuracy, sensitivity, and specificity values of 98.6%, 95.0%, and 99.0%, respectively, employing feature selection and an SVM classifier. Cheng and Dong. [79] achieved an accuracy of 95.50% employing a personalized features SVM. Mohanty et al. [16] detected and classified ventricular arrhythmias employing a cubic Support Vector Machine (SVM) and C4.5 classifiers, achieving Sen = 90.97%, Spe = 97.86%, and Acc = 97.02%. Li et al. [69] attained Sen = 98.4%, Spe = 98.0%, and Acc = 98.1% employing a genetic algorithm (GA) for feature selection and an SVM classifier. Xu et al. [80] attained high performance values (and Acc = 98.29%, Sen = 97.32% and Spe = 98.95%) utilizing adaptive variational and boosted CART.

The results of the Ht_TFR_CNN2 proposal in this work outperform those of other works in this group as well, achieving an accuracy of 99.61%, a sensitivity of 99.74%, and a specificity of 99.61%. Therefore, the benefits of using the Ht_TFR_CNN2 method in the classification procedure are evident. Ht_TFR_CNN2 can be successfully employed in the detection and classification of ventricular arrhythmia, as well as in the classification of shockable episodes. This illustrates that the fusion of CNN and TRF yields a resilient signal characterization, implying a potential and encouraging utilization of these attributes in Automated External Defibrillation (AED) and Implantable Cardioverter Defibrillation (ICD) treatments.

## 7. Application in a Real Clinical Setting

In real clinical settings, Artificial Intelligence (AI), specifically convolutional neural networks (CNNs), offers significant potential for enhancing patient care by detecting ventricular fibrillation (VF) in individuals at risk of cardiac arrest [81]. This approach facilitates swift VF identification through the rapid analysis of electrocardiograms (ECG) in emergency departments. AI models trained on diverse VF patterns can improve accuracy compared to manual interpretation by clinicians. AI-powered monitoring systems can continuously analyze ECG signals in critically ill patients, automatically alerting healthcare providers for VF detection, which is particularly valuable in intensive care units. Moreover, AI-assisted VF detection streamlines healthcare efficiency by helping prioritize patients based on urgency. Despite this promise, integrating AI-based VF detection requires overcoming challenges such as rigorous validation and regulatory approvals to ensure safety. Collaboration among clinicians, data scientists, and regulatory bodies is crucial for successful and safe AI implementation in healthcare. The aforementioned factors contribute to the efficacy of both Automated External Defibrillators (AEDs) and Implantable Cardioverter-Defibrillators (ICDs). In [82], a ’genetic’ programming (GP) model is employed to predict favorable defibrillation outcomes for patients with ventricular fibrillation (VF). In [82], the efficacy of a programmable automatic external cardioverter–defibrillator (AECD) is investigated within in-hospital cardiac arrest scenarios involving ventricular fibrillation (VF) and ventricular tachycardia (VT). Continuous research is necessary to refine AI algorithms, as demonstrated in this article, where the Pseudo Wigner–Ville (PWV) exhibited effective real-time classification without extensive computational time.

## 8. Conclusions

The accurate interpretation and differentiation of ventricular arrhythmias, such as VF and VT, are crucial for patient safety. In this paper, we introduced an innovative approach to feature extraction, seamlessly integrating RTF and CNN techniques, for VF detection. We observed a sensitivity rate of 98.16%, a specificity of 99.07%, and an accuracy of 98.91%; for ventricular tachycardia (VT), the sensitivity was noted at 90.45%, the specificity was 99.73%, and the accuracy was 99.09%; for normal sinus rhythms, the sensitivity was 99.34%, the specificity was 98.35%, and the accuracy was 98.89%; finally, for other rhythms, the sensitivity was 96.98%, the specificity was 99.68%, and the accuracy was 99.11%. Moreover, this study showcases an impressively high accuracy of 99.61%, with a sensitivity of 99.23% and a specificity of 99.74%, effectively discerning between shockable (VT/VF) and non-shockable rhythms.

The application of this innovative approach yields slightly or significantly improved results compared to previous comparable works using the Pseudo-Wigner–Ville t-f representation and a diverse range of CNNs. This indicates that the benefits of our methodology are independent of the classifier used. Additionally, our proposed methodology provides real-time detection of VF with low computational time, effectively differentiating it from other cardiac pathologies. This significantly enhances the accuracy of diagnosing patients experiencing these arrhythmias.

It is worth noting that these powerful results were achieved without the need for the preselection of episodes. Based on our findings, we conclude that this technique can be successfully applied to both the detection and classification of ventricular arrhythmia, including shockable rhythms. Moreover, it offers valuable features that facilitate the classification task. Despite the higher computational complexity during training, this technique has the potential to yield superior results not only in the field of ventricular arrhythmia detection but also in various bioengineering applications that currently involve a stage of feature selection and extraction prior to classification.

## Figures and Tables

**Table 1:** Details concerning the proposed CNN1 and CNN2 architecture.

Model	CNN1		
Layer	Kernel Size	Filter Number	#Parameters
FC1	512	-	16589312
FC2	256	-	131328
Softmax	4	-	1285
**Model**	CNN2		
Layer	Kernel Size	Filter Number	#Parameters
Conv1	3 × 3	32	320
Max Pooling1	4 × 4	-	0
Conv2	3 × 3	64	18496
Max Pooling2	4 × 4	-	0
FC1	128	-	991360
FC2	256	-	33024
Softmax	4	-	1028

**Table 2:** Results achieved for the classification of the Normal class during testing.

Class	Normal						
**Algorithms**	**Sensitivity (%)**	**Specificity (%)**				**Accuracy (%)**	**F Score (%)**
	**Normal**	**Global**	**VF**	**VT**	**Other**	**Total**	**Total**
Ht_TFR_CNN1 (Epochs = 50)	89.70	98.57	99.53	99.48	97.73	98.76	93.92
Ht_TFR_CNN1 (Epochs = 100)	99.29	98.62	98.88	99.33	98.03	98.91	98.95
Ht_TFR_CNN2 (Epochs = 100)	99.34	98.35	99.59	99.83	99.59	98.89	98.84
TFR_CNN1 (Epochs = 50)	98.70	98.59	99.46	98.73	97.73	98.65	98.64

**Table 3:** Results achieved for the classification of the Other class during testing.

Class	Other						
**Algorithms**	**Sensitivity (%)**	**Specificity (%)**				**Accuracy (%)**	**F Score (%)**
	**Other**	**Global**	**VT**	**Normal**	**VF**	**Total**	**Total**
Ht_TFR_CNN1 (Epochs = 50)	97.24	99.41	99.82	99.29	99.65	98.95	98.31
Ht_TFR_CNN1 (Epochs = 100)	97.74	99.62	99.83	99.60	99.58	99.22	98.67
Ht_TFR_CNN2 (Epochs = 100)	96.98	99.68	99.96	99.61	99.79	99.11	98.31
TFR_CNN1 (Epochs = 50)	97.24	99.47	100	99.33	99.73	98.98	98.34

**Table 4:** Results achieved for the classification of the *VT* class during testing.

Class	VT						
**Algorithms**	**Sensitivity (%)**	**Specificity (%)**				**Accuracy (%)**	**F Score (%)**
	**VT**	**Global**	**VF**	**Other**	**Normal**	**Total**	**Total**
Ht_TFR_CNN1 (Epochs = 50)	89.70	99.70	96.71	99.84	99.94	99.00	94.43
Ht_TFR_CNN1 (Epochs = 100)	92.70	99.53	97.78	99.94	99.92	99.06	95.99
Ht_TFR_CNN2 (Epochs = 100)	90.45	99.73	96.92	99.94	99.98	99.09	94.86
TFR_CNN1 (Epochs = 50)	95.84	97.19	98.55	99.84	99.84	97.90	96.51

**Table 5:** Results achieved for the classification of the VF class during testing.

Class	VF						
**Algorithms**	**Sensitivity (%)**	**Specificity (%)**				**Accuracy (%)**	**F Score (%)**
	**VF**	**Global**	**VT**	**Other**	**Normal**	**Total**	**Total**
Ht_TFR_CNN1 (Epochs = 50)	98.04	98.94	90.96	99.64	99.68	98.77	98.48
Ht_TFR_CNN1 (Epochs = 100)	96.44	99.28	94.01	99.74	99.76	98.75	97.83
Ht_TFR_CNN2 (Epochs = 100)	98.16	99.07	91.56	99.74	99.83	98.91	98.61
TFR_CNN1 (Epochs = 50)	85.88	99.30	96.58	99.64	99.52	96.82	92.10

**Table 6:** Results obtained for the classification of the Normal class in testing.

Class	Normal						
**Techniques**	**Sensitivity (%)**	**Specificity (%)**				**Accuracy (%)**	**F Score (%)**
	**Normal**	**Global**	**VF**	**VT**	**Other**	**Total**	**Total**
Ht_TFR_CNN1 (Epochs = 100)	99.29	98.62	98.88	99.33	98.03	98.91	98.95
Ht_TFR_CNN2 (Epochs = 100)	99.34	98.35	99.59	99.83	99.59	98.89	98.84
InceptionV3 (Epochs = 6)	77.99	99.65	99.92	39.30	99.32	87.17	87.49
MobilNet (Epochs = 6)	79.42	99.44	99.08	99.36	99.64	88.39	88.30
VGGnet (Epochs = 6)	96.61	98.32	97.97	100	98.59	97.39	97.45
AlexNet (Epochs = 6)	99.43	97.29	98.69	100	95.83	98.45	98.34

**Table 7:** Results obtained for the classification of the Other class in testing.

Class	Other						
**Techniques**	**Sensitivity (%)**	**Specificity (%)**				**Accuracy (%)**	**F Score (%)**
	**Other**	**Global**	**VT**	**Normal**	**VF**	**Total**	**Total**
Ht_TFR_CNN1 (Epochs = 100)	97.74	99.62	99.83	99.60	99.58	99.22	98.67
Ht_TFR_CNN2 (Epochs = 100)	96.98	99.68	99.96	99.61	99.79	99.11	98.31
InceptionV3 (Epochs = 6)	88.42	99.81	100	99.72	100	96.96	93.77
MobilNet (Epochs = 6)	99.64	85.08	98.41	79.60	97.68	88.21	91.78
VGGnet (Epochs = 6)	98.54	97.57	100	96.74	99.26	97.39	98.05
AlexNet (Epochs = 6)	95.78	99.57	100	99.58	99.40	98.77	97.63

**Table 8:** Results obtained for the classification of the VT class in testing.

Class	VT						
**Techniques**	**Sensitivity (%)**	**Specificity (%)**				**Accuracy (%)**	**F Score (%)**
	**VT**	**Global**	**VF**	**Other**	**Normal**	**Total**	**Total**
Ht_TFR_CNN1 (Epochs = 100)	92.70	99.53	97.78	99.94	99.92	99.06	95.99
HT_TFR_CNN2 (Epochs = 100)	90.45	99.73	96.92	99.94	99.98	99.09	94.86
InceptionV3 (Epochs = 6)	98.15	83.55	99.11	99.04	80.18	84.59	90.26
MobilNet (Epochs = 6)	95.53	97.66	98.89	100	99.90	97.49	96.58
VGGnet (Epochs = 6)	90.15	99.15	97.07	99.94	100	98.77	94.43
AlexNet (Epochs = 6)	91.84	99.47	97.54	99.94	100	98.94	95.50

**Table 9:** Results obtained for the classification of the VF class in testing.

Class	VF						
**Techniques**	**Sensitivity (%)**	**Specificity (%)**				**Accuracy (%)**	**F Score (%)**
	**VF**	**Global**	**VT**	**Other**	**Normal**	**Total**	**Total**
Ht_TFR_CNN1 (Epochs = 100)	96.44	99.28	94.01	99.74	99.76	98.75	97.83
Ht_TFR_CNN2 (Epochs = 100)	98.16	99.07	91.56	99.74	99.83	98.91	98.61
InceptionV3 (Epochs = 6)	77.28	94.90	98.15	89.72	96.86	91.28	85.18
MobilNet (Epochs = 6)	86.97	99.62	97.33	100	99.80	97.01	92.86
VGGnet (Epochs = 6)	93.34	99.25	92.28	100	99.85	98.14	96.20
AlexNet (Epochs = 6)	95.58	99.34	93.42	100	99.84	98.64	97.42

**Table 10:** Comparison of proposed CNN architecture for applications in detecting Normal, Other, VT, and VF classes with other techniques.

Class	VF			VT			Other			Normal			Data Base
**Techniques**	**Sens (%)**	**Spe (%)**	**Acc (%)**	**Sens (%)**	**Spe (%)**	**Acc (%)**	**Sens (%)**	**Spe (%)**	**Acc (%)**	**Sens (%)**	**Spe (%)**	**Acc (%)**	
This work, Ht_TFR_CNN1 (Epochs = 50)	98.04	98.94	98.77	89.7	99.70	99	97.24	99.41	98.95	89.7	98.57	98.76	MITBIH, AHA
This work, Ht_TFR_CNN1 (Epochs = 100)	96.44	99.28	98.75	92.70	99.53	99.06	97.74	99.62	99.22	99.29	98.62	98.91	MITBIH, AHA
This work, Ht_TFR_CNN2 (Epochs = 100)	98.16	99.07	98.91	90.45	99.73	99.09	96.98	99.68	99.11	99.34	98.35	98.89	MITBIH, AHA
This work, InceptionV3 (Epochs = 6)	77.28	94.9	91.28	98.15	83.55	84.59	88.42	99.81	96.96	77.99	99.65	87.17	MITBIH, AHA
This work, MobilNet (Epochs = 6)	86.97	99.62	97.01	95.53	97.66	97.49	99.64	85.08	88.21	79.42	99.44	88.39	MITBIH, AHA
This work, VGGnet (Epochs = 6)	93.34	99.25	98.14	90.15	99.15	98.77	98.54	97.57	97.39	96.61	98.32	97.39	MITBIH, AHA
This work, AlexNet (Epochs = 6)	95.58	99.34	98.64	91.84	99.47	98.94	95.78	99.57	98.77	99.43	97.29	98.45	MITBIH, AHA
[58] SSVR, TFR	91	97		92.8	98.7		92.3	99.2		96.6	96.3		MITBIH, AHA
[58] BAGG, TFR	95.2	96.4		88.8	99.7		88.6	99.8		96.6	94.1		MITBIH, AHA
[58] I2-RLR and TFR	89.6	96.7		91	98.1		92.5	98.1		94.9	96.4		MITBIH, AHA
[58] ANNC and TFR	92.8	97		91.8	98.7		92.9	99		96.2	96.7		MITBIH, AHA
[66] TCSC algorithm	80.97	98.51	98.14										MITBIH, CUDB
[67] Chaotic based			88.6										MITBIH, CCU
[68] SVM and FS	91.9	97.1	96.8										MITBIH, CUDB
[69] SVM and Genetic algorithm	98.4	98	96.3										CUDB, AHA
[70] SVM and EMD	99.99	98.4	99.19										MITBIH, CUDB
[71] CNN neural network	56.44	98.19	97.88										MITBIH, CUDB
[72] EMD and Lempel-Ziv	98.15	96.01		96.01	98.15								MITBIH, CUDB
[73] TDA	97.07	99.25	98.68	92.72	99.53	99.05	97.43	99.54	99.09	99.05	98.45	98.76	MITBIH, AHA
[73] PDI	84.34	96.77	94.26	82.25	98.53	97.38	92.86	97.15	96.19	93.09	92.14	92.65	MITBIH, AHA
[74] App Entropy and EMD	90.47	91.66	91.17	90.62	91.11	90.8							MITBIH
[75] Approximated entropy	97.98	97.03		97.03	97.98								MITBIH, CUDB

**Table 11:** Comparison of proposed CNN architecture for applications in detecting ventricular fibrillation and tachycardia with other techniques.

Class	Shockable (VT+VF)			Data Base
**Technique**	**Sensitivity (%)**	**Specificity (%)**	**Accuracy (%)**	
This work, Ht_TFR_CNN1	98.53	99.69	99.39	MITBIH, AHA
This work, Ht_TFR_CNN2	99.23	99.74	99.61	MITBIH, AHA
[73] TDA	99.03	99.67	99.51	MITBIH, AHA
[73] PDI	89.63	96.96	95.12	MITBIH, AHA
[76] CNN	95.32	91.04	93.2	MITDB, CUDB, VFDB
[14] VMD with Random Forest	96.54	97.97	97.23	MITBIH, CUDB
[77] RNN			99.72	MITBIH
[78] CNN and IENN	98.6	98.9	98.8	MITBIH, AFDB
[68] FS and SVM	95	99	98.6	MITBIH, CUDB
[79] Personalized features SVM		95.6	95.5	MITBIH, CUDB, VFDB
[16] C4.5 classifier	90.97	97.86	97.02	MITBIH, CUDB
[69] SVM and bootstrap	98.4	98	98.1	MITBIH, AHA, CUDB
[80] Adaptive variational and boosted CART	97.32	98.95	98.29	MITBIH, CUDB

## Data Availability

Publicly available data in Physionet: http://physionet.org/.
